# Juvenile idiopathic arthritis (JIA): can an integrated medicine be possible?

**DOI:** 10.1186/1546-0096-9-S1-P195

**Published:** 2011-09-14

**Authors:** A Officioso, G Griso, C Storace, A Battagliese, R Carlomagno, M Alessio

**Affiliations:** 1 Department of Paediatrics, University Federico II, Naples, Italy

## Background

JIA means arthritis of unknown origin, lasting more than 6 weeks and that arise before 16 years of age. It is characterized by pain, swelling, stiffness and functional limitation of joints. The Multidimensional Self-Concept Scale assesses global self-concept and six context-dependent self-concept domains that are functionally and theoretically important in the social-emotional adjustment of youth and adolescents: Social, Competence, Affect, Academic, Family, and Physical.

## Aim

To evaluate the Self-concept and social-emotional functioning of adolescents with JIA after a global treatment including psychological support, physical therapy in addition to pharmacological therapy.

## Patients

30 patients aged 11 to 19 years, 15 of which are only evaluated during drug treatment (A) and 15 evaluated after two years of integrated care (B), more than medical care participated in a group of body psychotherapy, as well as its parents took part in a 2 years trial.

## Methods

Self-Concept and Self-Esteem assessment was performed at entry into the trial and after 2 year , by means of self-rating questionnaires.

## Results

Preliminary analysis of the results (table 1) shows that after 2 years patients receiving a global treatment experienced significant psychological improvement showing a higher self-esteem compared to the other group.

**Table 1:** 

	Social	Competence	Affect	Academic	Family	Physical	Total
A	58,8±15,8	55,2±26,4	62,8±15,6	52,4 ±28,6	53,6±17,3	27±15,8	53,4 ±19,2
B	67,4±17,0	67,6±22,6	48,8±14,9	72±21,9	71±9	31,8±26,9	64,2±16,0

## Conclusion

Our findings suggest that the treatment of arthritis need to be global in order to get some good results. The strength of this study, we believe that should be seen in the rheumatologist's ability to pass:a) the flexibility versus rigidity, b) drugs mixed with humanity,c) the ability to share the patient with other professionals

